# Construction of an lncRNA model for prognostic prediction of bladder cancer

**DOI:** 10.1186/s12920-022-01414-6

**Published:** 2022-12-14

**Authors:** Changlong Shi, Yifei Li, Enming Wan, Enchong Zhang, Li Sun

**Affiliations:** 1 Department of Urology, Huishan District People’s Hospital, Wuxi City, Jiangsu China; 2 Department of Second Urology, Shengjing Hospital of China Medical University, Shenyang, Liaoning China; 3 Department of Breast Surgery, Huishan District People’s Hospital, Wuxi City, Jiangsu China

**Keywords:** BC, lncRNA, Prognosis, TCGA

## Abstract

**Objective:**

We aimed to investigate the role and potential mechanisms of long non-coding RNAs (lncRNAs) in bladder cancer (BC), as well as determine their prognostic value.

**Methods:**

LncRNA expression data and clinical data from BC patients were downloaded from The Cancer Genome Atlas (TCGA) database. R software was used to carry out principal component analysis (PCA), differential analysis, and prognostic analysis. Lasso regression and multivariate Cox regression analyses were performed to identify potential prognostic genes. The expression of five identified genes and their correlation with prognosis were verified using TCGA and GSE13507 datasets. In addition, quantitative real-time polymerase chain reaction (qRT-PCR) was used to confirm the expression of these five genes in cell lines (two human BC cell lines and one human bladder epithelial cell line) and tissues (84 pairs of BC tissues and the corresponding paracancerous tissues). Risk scores that had been generated from the five genes and their prognostic ability were assessed by receiver operating characteristic (ROC) and Kaplan–Meier (KM) curves. Co-expressed genes were screened by WGCNA and analyzed by GO and KEGG, while functional enrichment and immune infiltration analyses were performed using STRING (https://cn.string-db.org/) and TIMER2.0 (http://timer.cistrome.org/) online tools, respectively.

**Results:**

CYP4F8, FAR2P1, LINC01518, LINC01764, and DTNA were identified as potential prognostic genes. We found that these five genes were differentially expressed in BC tissue, as well as in BC cell lines, and were significantly correlated with the prognosis of BC patients. KM analysis considering risk scores as independent parameters revealed differences in overall survival (OS) by subgroups. The ROC curve revealed that a combined model consisting of all five genes had good predictive ability at 1, 3, and 5 years. GO and KEGG analyses of 567 co-expressed genes revealed that these genes were significantly associated with muscle function.

**Conclusion:**

LncRNAs can be good predictors of BC development and prognosis, and may act as potential tumor markers and therapeutic targets that may be beneficial in helping clinicians decide the most effective treatment strategies.

## Introduction

Bladder cancer (BC) is ranked tenth among the most common cancers worldwide and ninth among cancer deaths accounting for approximately 573,000 new cases and 213,000 deaths in 2020 [1]. By 2022, the incidence and mortality of BC in China is predicted to be higher than in developed countries such as the United States [2]. Therefore, there is a critical need to develop novel non-invasive markers to improve the diagnosis and treatment of BC.

In the human genome more than 98% of genes do not encode proteins, and are termed non-coding RNAs (ncRNAs) [3]. Long non-coding RNAs (lncRNAs) have been identified as RNA transcripts containing more than 200 nucleotides [4]. LncRNAs are not only the predominant type of ncRNA, but are also considered to be essential regulators of a range of biological processes [5, 6].

The Cancer Genome Atlas (TCGA) database was started in 2006, and now contains data on 33 types of cancer, as well as the molecular typing of more than 20,000 primary cancers. The TCGA database has had a profound impact on global oncological research, and will be important in identifying new targets associated with BC.

In this study, we identified five novel genes by screening BC data in the TCGA database. By examining their expression profiles and prognostic relevance, as well as performing functional enrichment analysis, we determined the potential biological characteristics of these five genes.

## Methods

### Source and processing of the dataset

RNA data corresponding to 411 BC tissue and 19 paraneoplastic tissue samples were obtained from TCGA using GENCODE software. Analysis using the RTCGA clinical package in R software revealed 409 cases of uroepithelial carcinoma, one case of squamous carcinoma, and one case of adenocarcinoma in the 411 samples. Of the 409 uroepithelial carcinomas, four were stage I, 131 were stage II, 141 were stage III, and 136 were stage IV. Of these, 21 were low-grade, and 388 were high-grade uroepithelial carcinomas. The biomaRt package was used to screen a total of 24,971 genes for PCA analysis, and the sva R package was used to remove batch effects. The GSE13507 dataset—comprising clinical data on 188 BC tissues (165 primary uroepithelial carcinomas and 23 recurrent carcinomas) and 68 paracancerous tissues was downloaded from the GEO database using the clusterProfiler package. Of the 165 uroepithelial carcinomas, 103 were stage I, 26 were stage II, 28 were stage III and 8 were stage IV. In total, the study comprised 105 low-grade and 60 high-grade uroepithelial carcinomas.

### Selection and validation of differential genes and prognosis-related genes

Differential expression of the 24,971 genes was analyzed using the limma package, and further screened for significantly upregulated and downregulated genes using the DESeq2 package (|Log2FoldChange|> 3.5, *P* < 0.05). In addition, univariate Cox analysis (Hazard Ratio (HR) ≠ 1, *p* < 0.05) was performed using the survival package. Prognosis-related genes with HR > 1 were intersected with upregulated differentially expressed genes separately using the VennDiagram package,while prognosis-related genes with HR < 1 were intersected with downregulated differentially expressed genes. The Glmnet package was used to perform Lasso regression and tenfold cross-validation on the intersecting genes to determine the optimal lambda (*λ*) value for the minimum partial likelihood deviation to derive the validation genes. Multivariate Cox regression analysis was performed on the validated genes to identify the most relevant genes.

### Expression and prognostic analysis of the identified genes in the TCGA and GSE13507 databases

Differences between the identified gene expression levels in cancer and paracancer samples were analyzed in TCGA and GSE13507 datasets using the dplyr package. The median expression levels of the identified genes were used as the boundary, and Kaplan–Meier (KM) survival curves based on the survminer R package were used to compare differences in survival between the high and low expression groups of the identified genes in the two databases.

### Analysis of gene expression levels in bladder cells and tissues

Two human BC cell lines (T24 and 5637) and a human bladder epithelial cell line (SV-HUC-1) were obtained from Fu Heng Biologicals (Shanghai, China). T24 cells were cultured in DMEM containing 10% fetal bovine serum (FBS), while 5637 cells were cultured in RPMI 1640 containing 10% FBS. Cell lines were cultured at 37 °C in a 5% CO2 incubator. All of the above culture materials were obtained from Gibco. A total of 84 pairs of BC and paracancer specimens were selected from our previous collections. Among them, 36 pairs were muscle invasive BC (MIBC) and 48 pairs were non–muscle invasive BC (NMIBC). All specimens were placed in liquid nitrogen immediately after surgical excision and transferred to a − 80 °C freezer for long-term storage within 30 min. Total RNA was extracted from the cells and tissues using TRIzol. The cDNA was synthesized by reverse transcription using the PrimeScript RTTM Master Mix Takara (Biotech, Dalian, China) according to the manufacturer’s instructions. qRT-PCR was performed with SYBR Premix Ex TaqTM II to determine the expression levels of the five identified genes. Relative mRNA expression levels were calculated using the 2^−ΔΔCt^ method. *β*- actin was used as the control. The primer sequences are shown in Table 1.

**Table 1:** Primer sequences

Gene name	Primer sequences (5′ to 3′)
CYP4F8	Forward: CGAGTCATCCCCAAAGGGAA
	Reverse: GCAAAACAATCTCCGGCGTC
FAR2P1	Forward: ATCACAGCCCTCCAGGAGAT
	Reverse: CACACTGCGTTCCCTCTGAT
LINC01518	Forward: GTGGCCACCATGACAAGGAA
	Reverse: TTGGCCATGATCCCTTCTGC
LINC01764	Forward: CCTGTTTCCCTCTCCTGCAAT
	Reverse: AGCACCAGCTGACATGGTAT
DTNA	Forward: AGACACAGTACACACCAGGA
	Reverse: CCATGGCCTTCCGGATCAAA
*β*-actin	Forward: GACGAGGACCAGGTAAGCAATGAC
	Reverse: GACACCATCTGAGGAGAACGCATG

### Construction and evaluation of the risk score model

Risk score models were constructed based on the multivariate Cox regression data. Forest plots were drawn using the ggforest package. Risk factor linkage plots were drawn using the ggrisk software package. Based on the median risk score, patients were divided into high-risk and low-risk groups. KM survival curves were used to compare differences between high-risk and low-risk subgroups in terms of gender, age, grading, and stage. Time-dependent receiver operating characteristic (ROC) curves based on the survROC package were used to plot risk scores and compare the prognostic accuracy of each gene.

### Identification and functional analysis of co-expressed coding genes

The WGCNA software package was used to implement variance screening of the underlying data, analysis of variance between samples, removal of outlier samples, determination of soft thresholds, co-expression module mining, and calculation of correlations between genes and modules. Co-expressed genes of each module were exported and visualized by Cytoscape software. The co-expressed genes were de-duplicated and organized using the TCGA and MSigDB gene sets (c2.all.v7.5.1.entrez.gmt). GO and KEGG functional enrichment analyses were carried out using the clusterProfiler package in the TCGA and MSigDB gene sets (c2.all.v7.5.1.entrez.gmt).

### Key coding gene screening and immune infiltration analysis

Co-expressed genes were imported into the STRING online tool to determine protein interactions (screening criteria were scores > 0.4). The top 10 coding genes were obtained using the cytoHubba plugin in Cytoscape software and re-imported into STRING to analyze their functional properties. Immune infiltration analysis was carried out using the TIMER2.0 online tool.

## Results

### Screening of differential genes and prognosis-related genes

**Fig. 1:**
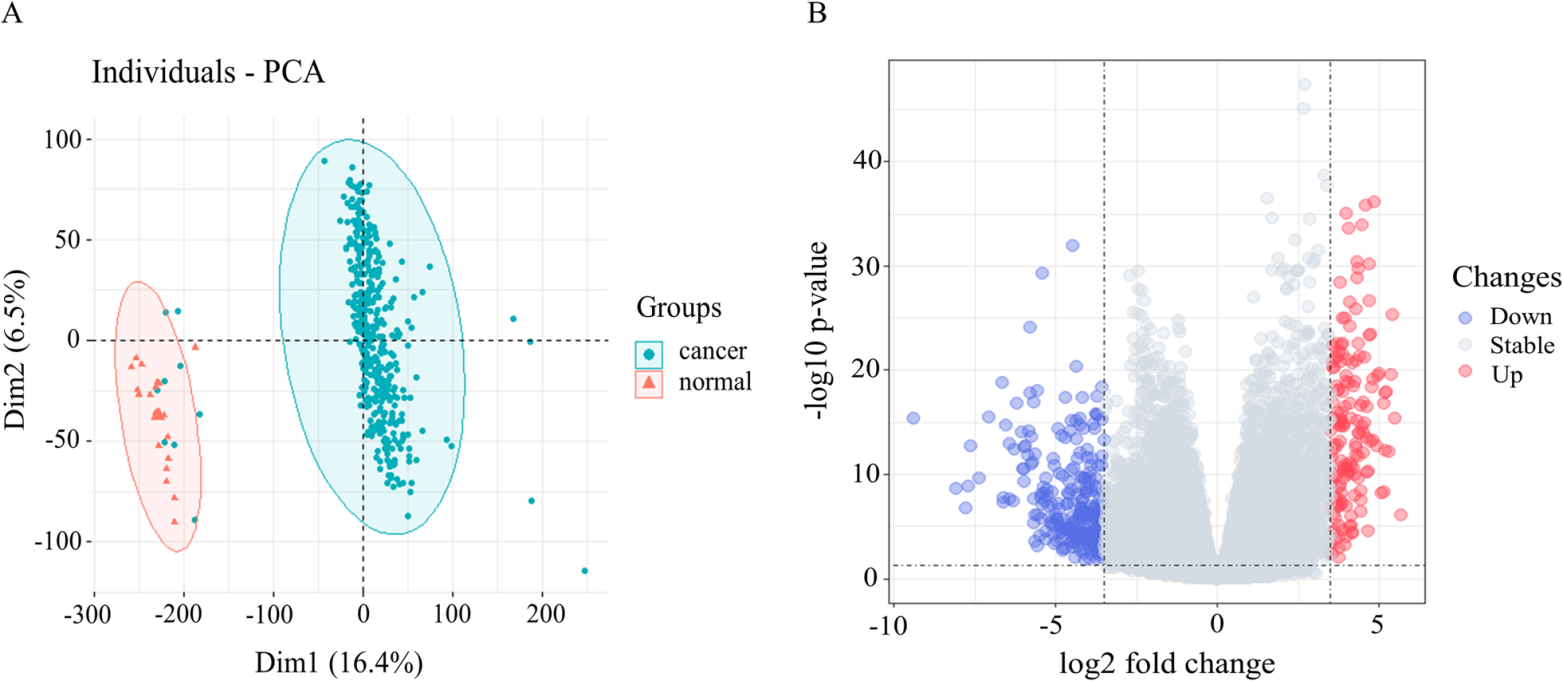
PCA analysis and differential gene screening. **A**: PCA analysis showed significant differences in transcriptome levels between tumor and paraneoplastic tissues, while there was no batch effect. Blue dots represent tumor clusters and red dots represent paracancerous clusters. **B**: Volcano map of differentially expressed genes in BC identifying 426 differentially expressed genes. Red dots represent upregulated genes (159). Blue dots represent downregulated genes (267). The figure was made by *R* software (*R*, version 4.1.2, University of Auckland, New Zealand)

A total of 24,971 coding genes were screened in the TCGA database. PCA analysis revealed significant differences in expression levels between cancer and paraneoplastic tissues with no significant crossover (Fig. 1A). Differential analysis identified 159 upregulated and 267 downregulated genes (Fig. 1B, https://doi.org/10.6084/m9.figshare.20055800.v1). Prognostic analysis identified 2421 genes with HR > 1, and 2820 genes with HR < 1.

### Identification and validation of genes

**Fig. 2:**
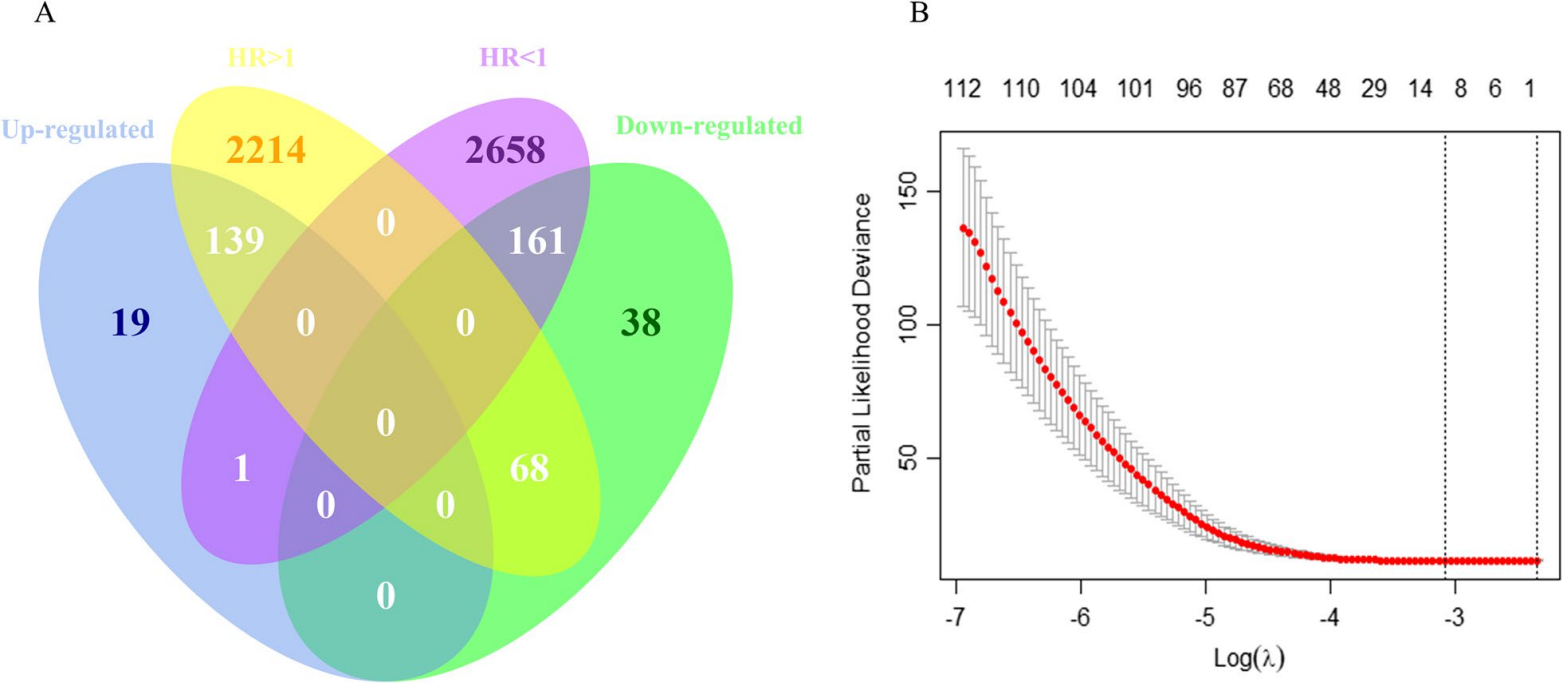
Final gene screening. **A**: Venn diagram showing the intersection between differentially expressed genes and prognostic-related genes. Of a total of 300 genes, 139 genes intersected with HR > 1 for upregulation and 161 genes intersected with HR < 1 for downregulation. **B**: Lasso regression with cross-validation yielded a total of 12 genes associated with prognosis. The figure was made by *R* software (*R*, version 4.1.2, University of Auckland, New Zealand)

**Fig. 3:**
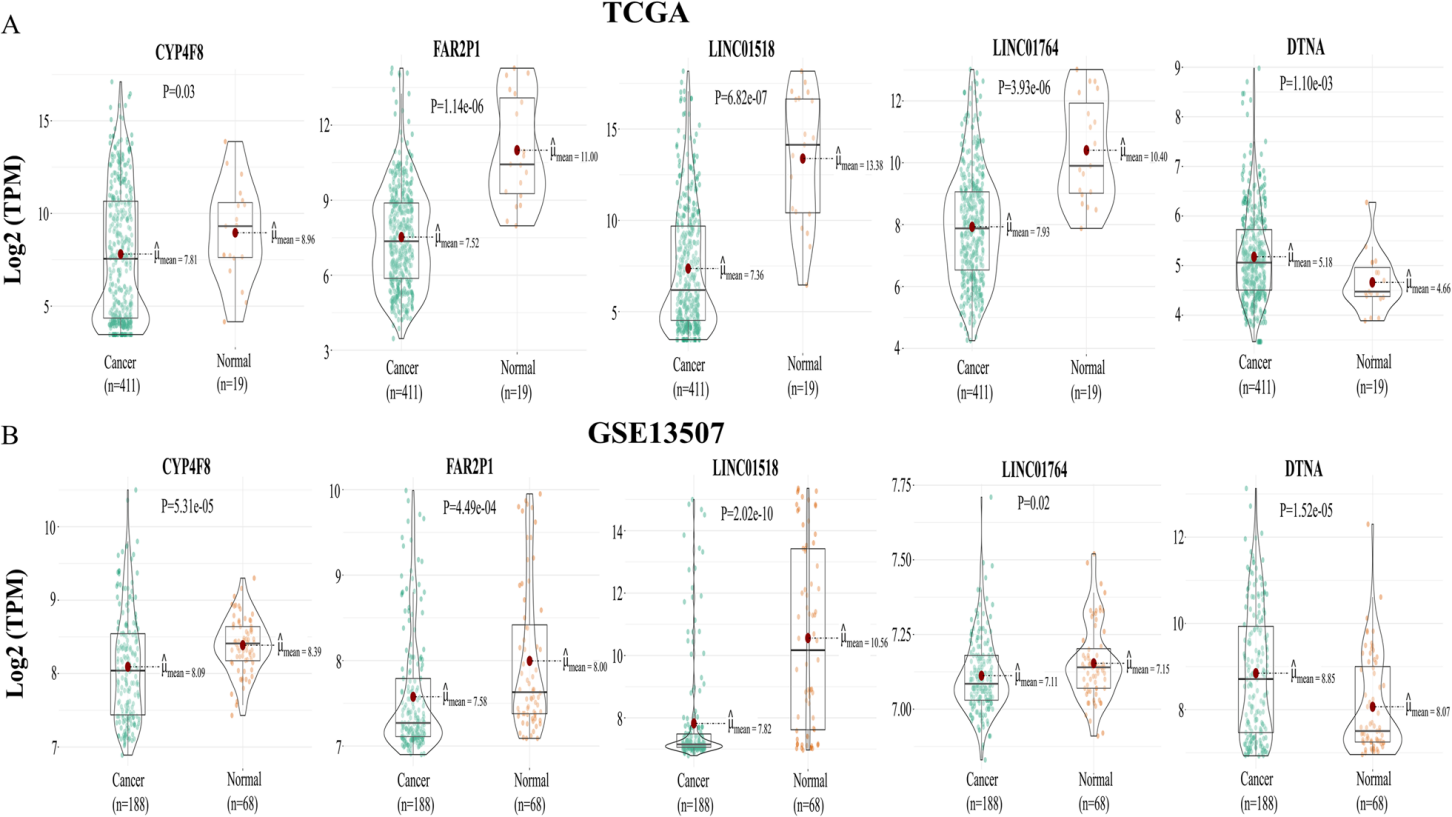
Verification of the identified gene expression levels. **A**: In the TCGA database, the expression levels of CYP4F8, FAR2P1, LINC01518, and LINC01764 genes were lower in BC tissues than in normal tissues, while elevated DTNA expression levels were found in BC tissues compared to normal tissues. **B**: In the GSE13507 dataset, CYP4F8, FAR2P1, LINC01518, and LINC01764 expression levels were lower in BC tissues than normal tissues, while DTNA expression levels were higher in BC samples than normal tissues. This figure was made by R software (R, version 4.1.2, University of Auckland, New Zealand)

**Fig. 4:**
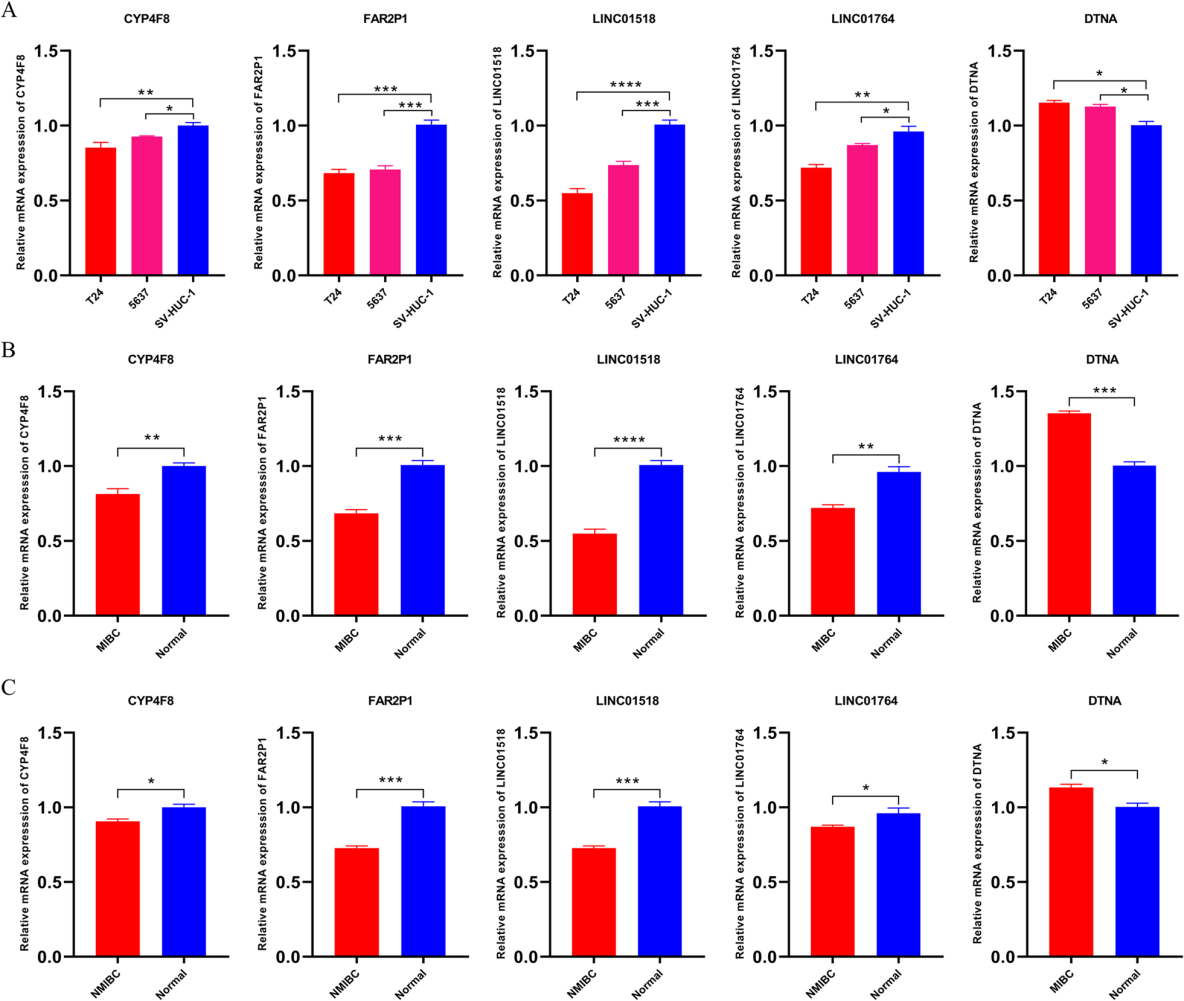
Verification of the identified gene expression levels. **A**: The expression levels of the five identified genes were detected by qRT-PCR in T24, 5637 and SV-HUC-1 cells. **B**: The expression levels of CYP4F8, FAR2P1, LINC01518, and LINC0176 were lower in MIBC tissues than those observed in paraneoplastic tissues, while DTNA expression levels were higher in MIBC tissues than in paraneoplastic tissues. **C**: The expression levels of CYP4F8, FAR2P1, LINC01518, and LINC0176 were lower in NMIBC tissues than those observed in paraneoplastic tissues, while the DTNA expression level was higher in NMIBC tissues than in paraneoplastic tissues. *β*-actin was used as the control. **** *P* < 0.001, *** *P* < 0.005, ** *P* < 0.01, * *P* < 0.05. This figure was made by GraphPad Prism software (GraphPad Prism, version 8.0.2, GraphPad Software, San Diego, California, USA)

**Fig. 5:**
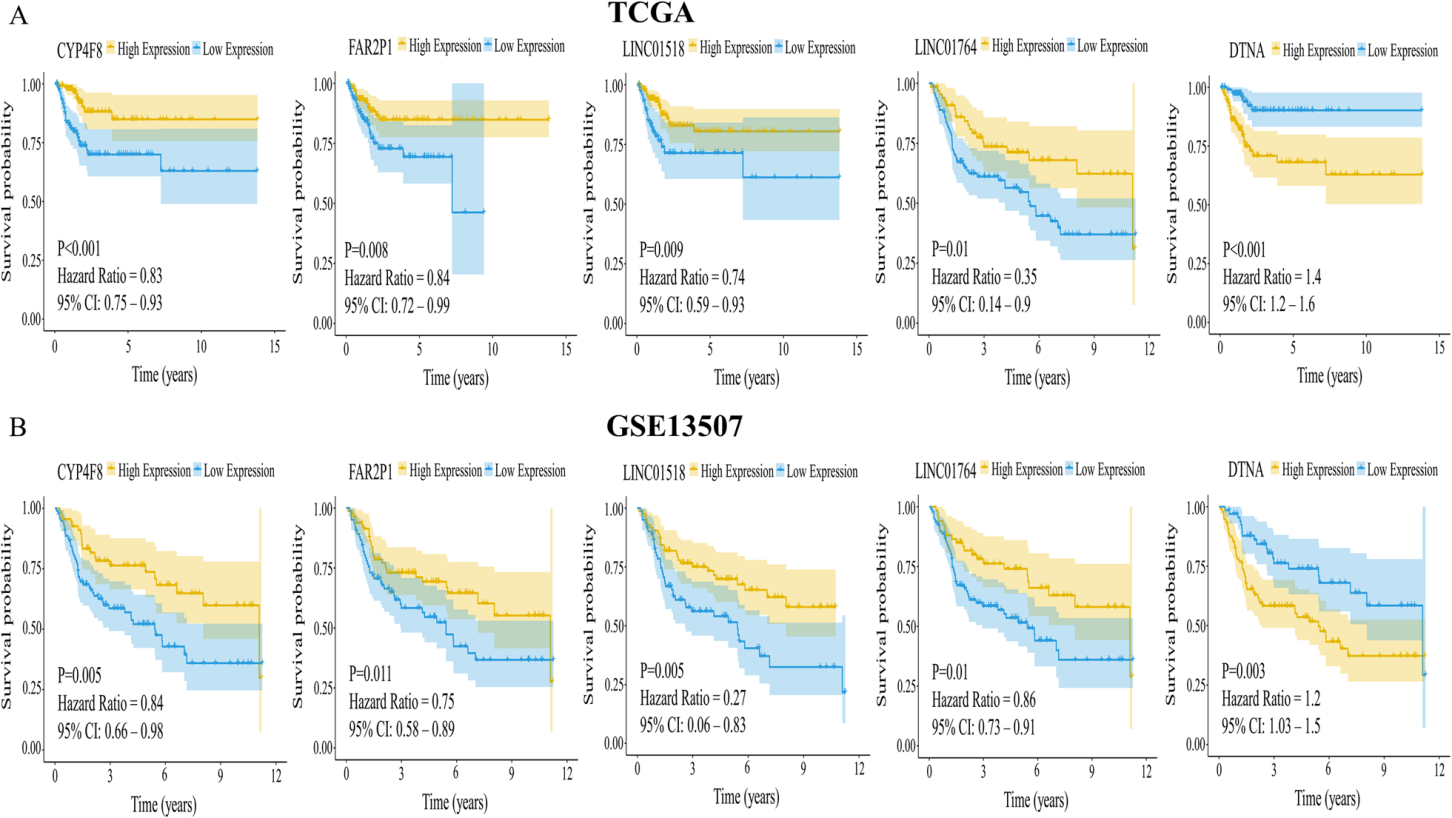
Validation of the association between the identified genes and patient prognosis. **A**: In the TCGA database, high DTNA expression levels together with low CYP4F8, FAR2P1, LINC01518 and LINC01764 expression levels were associated with poor prognosis in patients with BC. **B**: In the GSE13507 dataset, high expression of DTNA together with low expression of CYP4F8, FAR2P1, LINC01518, and LINC01764 was associated with poor prognosis in BC patients. The figure was made by R software (R, version 4.1.2, University of Auckland, New Zealand)

A total of 300 overlapping genes were identified by intersecting the prognostic-related genes with the differentially expressed genes (Fig. 2A). A total of 12 genes were identified after subjecting the overlapping genes to a tenfold validated Lasso regression analysis (Fig. 2B). Following Cox univariate and multivariate analyses of these 12 genes, the following five genes were identified: CYP4F8, FAR2P1, LINC01518, LINC01764, and DTNA (Table 2). Analysis of the TCGA database revealed that the expression levels of CYP4F8, FAR2P1, LINC01518, and LINC01764 were lower in cancerous than paracancerous tissues. In contrast, elevated DTNA expression levels were found in cancerous tissues compared to paracancerous tissues (Fig. 3A). Similar results were obtained using the GSE13507 dataset (Fig. 3B). Further validation by qRT-PCR revealed that the expression levels of CYP4F8, FAR2P1, LINC01518, LINC0176 in T24 and 5637 cell lines were lower than those observed in SV-HUC-1 cells, while DTNA expression levels were higher in T24 and 5637 cell lines compared to SV-HUC-1 cells (Fig. 4A). The expression levels of CYP4F8, FAR2P1, LINC01518, and LINC0176 in MIBC tissues were lower than those observed in paraneoplastic tissues, while the expression levels of DTNA in MIBC tissues were higher than those in paraneoplastic tissues (Fig. 4B). The validation results in NMIBC tissues were consistent with those in MIBC tissues (Fig. 4C). Using the median expression of five genes as cutoff values, we found that high expression of DTNA and low expression of CYP4F8, FAR2P1, LINC01518, and LINC01764 were associated with poor prognoses in BC patients (Fig. 5A–B).

**Table 2:** Results of univariate and multivariate COX regression analysis

Gene	Univariate analysis			Multivariate analysis		
	HR	95% CI	*P*-value	HR	95% CI	*P*-value
CYP4F8	0.216	0.109–0.428	< 0.001	1.183	0.947–1.462	0.131
FAR2P1	0.173	0.042–0.719	0.016	0.741	0.537–0.966	0.039
LINC01764	0.237	0.120–0.469	< 0.001	0.786	0.658–0.957	0.015
LINC01518	0.085	0.012–0.621	0.015	0.512	0.274–0.974	0.042
DTNA	4.190	1.845–9.513	0.001	1.144	1.033–1.264	0.001
AC110285.6	0.275	0.145–0.520	< 0.001			
AC245100.6	0.133	0.018–0.968	0.046			
PSG5	0.293	0.090–0.951	0.041			
AC009102.2	2.784	1.409–5.499	0.003			
AKAP6	3.649	1.859–7.165	< 0.001			
FGF6	2.828	1.403–5.697	0.004			
PCOLCE2	3.836	2.044–7.197	< 0.001

### Construction and evaluation of a 5-gene prognostic model

**Fig. 6:**
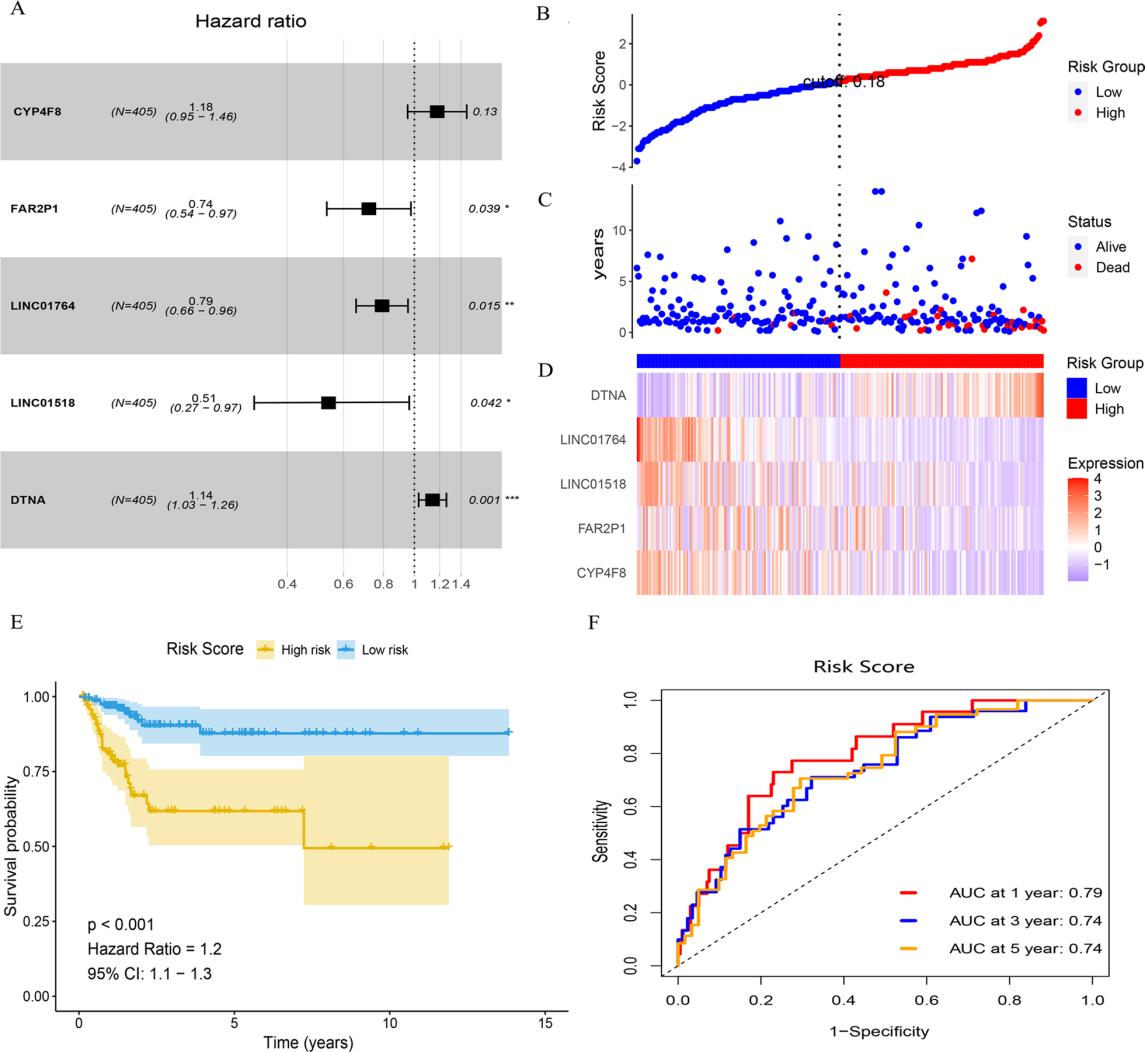
Identification of independent prognostic factors by Cox analysis, and construction and evaluation of risk models. **A**: Forest plot of multivariate Cox regression. **B**: Risk score curve. **C**: Increased number of patient deaths were associated with an increasing risk score. **D**: Heat map of the five identified gene expression profiles. **E**: KM curve based on risk score. A worse OS was observed in the high-risk group compared to the low-risk group. **F**: ROC curve showing the prognostic data for the five combined genes at 1, 3, and 5 years. The figure was made by R software (R, version 4.1.2, University of Auckland, New Zealand)

**Fig. 7:**
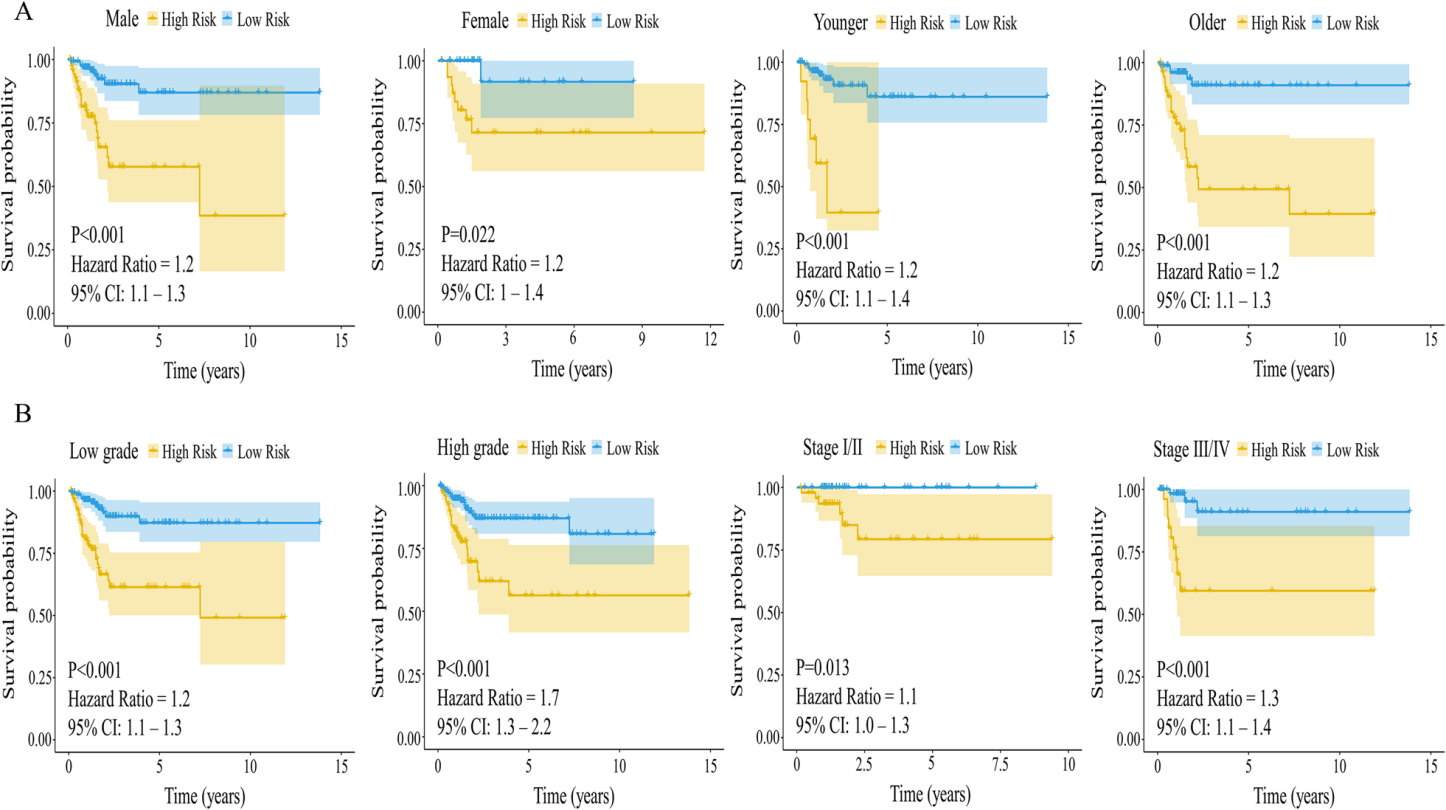
Risk model assessment of the different subgroups of the five identified genes. **A**: KM survival analysis curve of risk scoring model for sex and age subgroups. **B**: KM survival analysis curve of risk scoring model for grade and stage subgroups. The figure was made by R software (R, version 4.1.2, University of Auckland, New Zealand)

The forest plot based on the Cox multivariate regression data is shown in Fig. 6A (https://doi.org/10.6084/m9.figshare.20055965.v1). The following prognostic model risk scores were obtained:1.2563*DTNA- 0.9114*CYP4F8-0.8783*FAR2P1-0.8659*LINC01518-0.6284*LINC01764. Next, the risk score was calculated for each patient using the survminer and ggrisk package cutoff values. Patients were then divided into high-risk and low-risk groups (Fig. 6B, https://doi.org/10.6084/m9.figshare.20055965.v1). We found that an increased number of patient deaths was associated with a higher risk score in the corresponding A-plot (Fig. 6C). The heat map of the five gene expression profiles revealed that DTNA was expressed at high levels, while CYP4F8, FAR2P1, LINC01518, and LINC01764 were expressed at low levels (Fig. 6D). KM survival curves showed that the OS was worse in the high-risk group compared to the low-risk group (Fig. 6E, https://doi.org/10.6084/m9.figshare.20055971.v1). ROC analysis indicated that a combined model consisting of all five genes had good predictive ability at 1 year (ACU value = 0.79), 3 years (ACU value = 0.74) and 5 years (ACU value = 0.74) (Fig. 6F, https://doi.org/10.6084/m9.figshare.20055974.v1). KM survival analysis based on the 5-gene risk score models showed that relative to the low-risk group, OS was worse in the high-risk group in each subgroup (Fig. 7A-B, https://doi.org/10.6084/m9.figshare.20055980.v1).

### Potential biological functions of the five identified genes

**Fig. 8:**
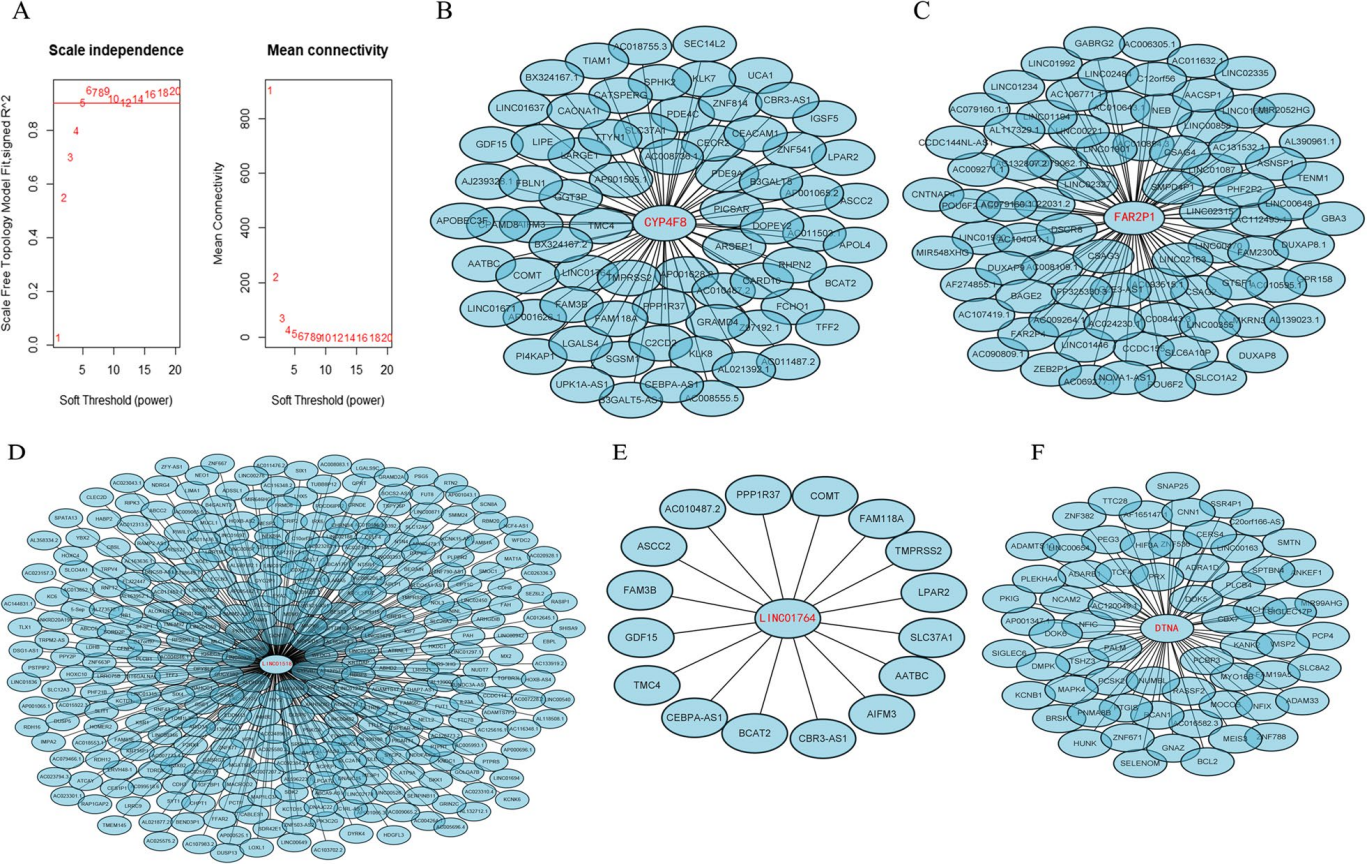
Establishment of co-expression network under WGCNA analysis. **A**: The soft threshold was determined based on a goodness-of-fit index. **B**: Coding genes co-expressed with CYP4F8. **C**: Coding genes co-expressed with FAR2P1. **D**: Coding genes co-expressed with LINC01518. **E**: Coding genes co-expressed with LINC01764. **F**: Coding genes co-expressed with DTNA. The figure was made by R software (R, version 4.1.2, University of Auckland, New Zealand) and Cytoscape software (Cytoscape, version 3.9.1, National Resource for Network Biology, US)

**Fig. 9:**
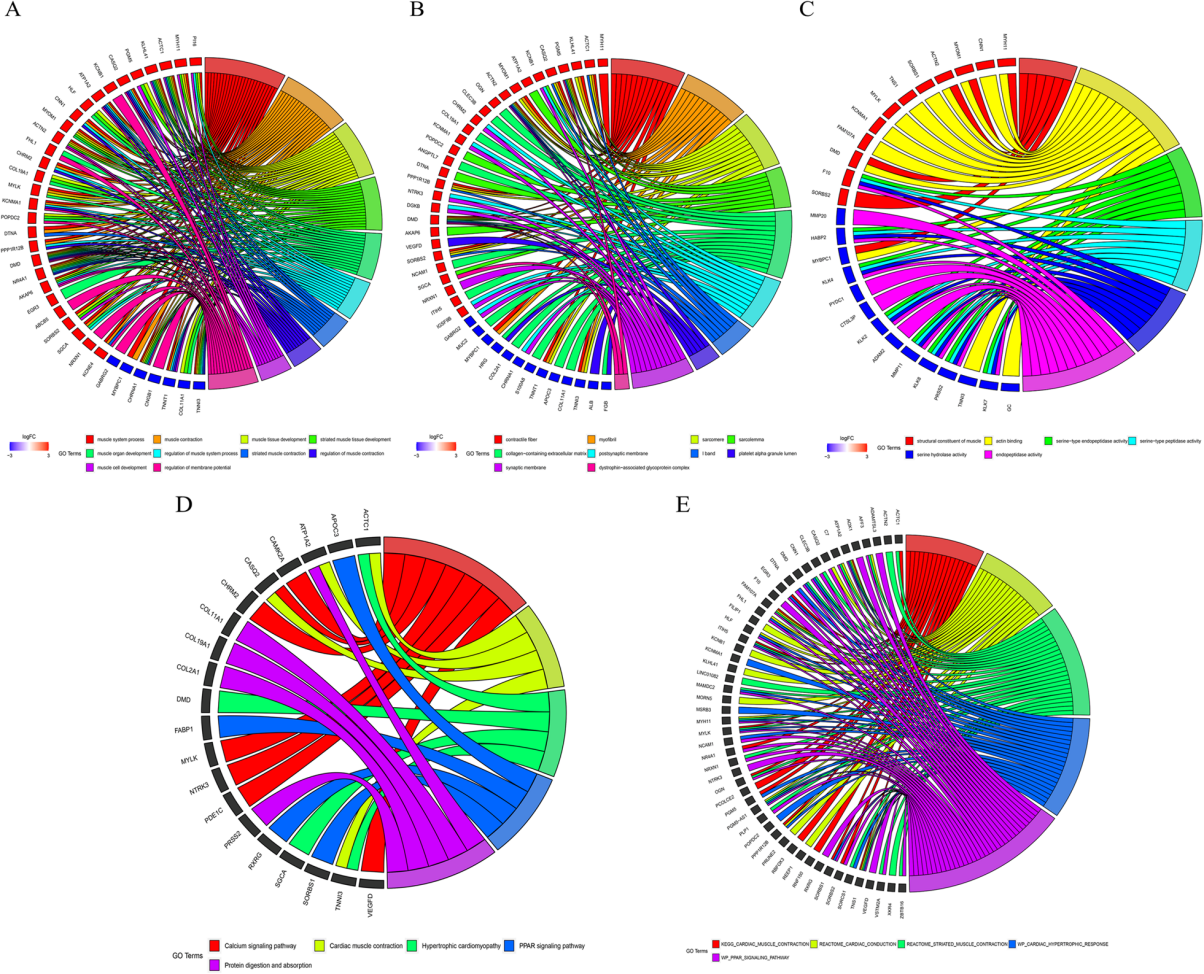
Functional analysis of prognosis-related genes. **A**: Biological processes in GO analysis. **B**: Cytological components in GO analysis. **C**: Molecular biological functions in GO analysis. **D**: KEGG analysis [34]. **E**: Analysis of MSigDB gene set. The figure was made by R software (R, version 4.1.2, University of Auckland, New Zealand)

**Fig. 10:**
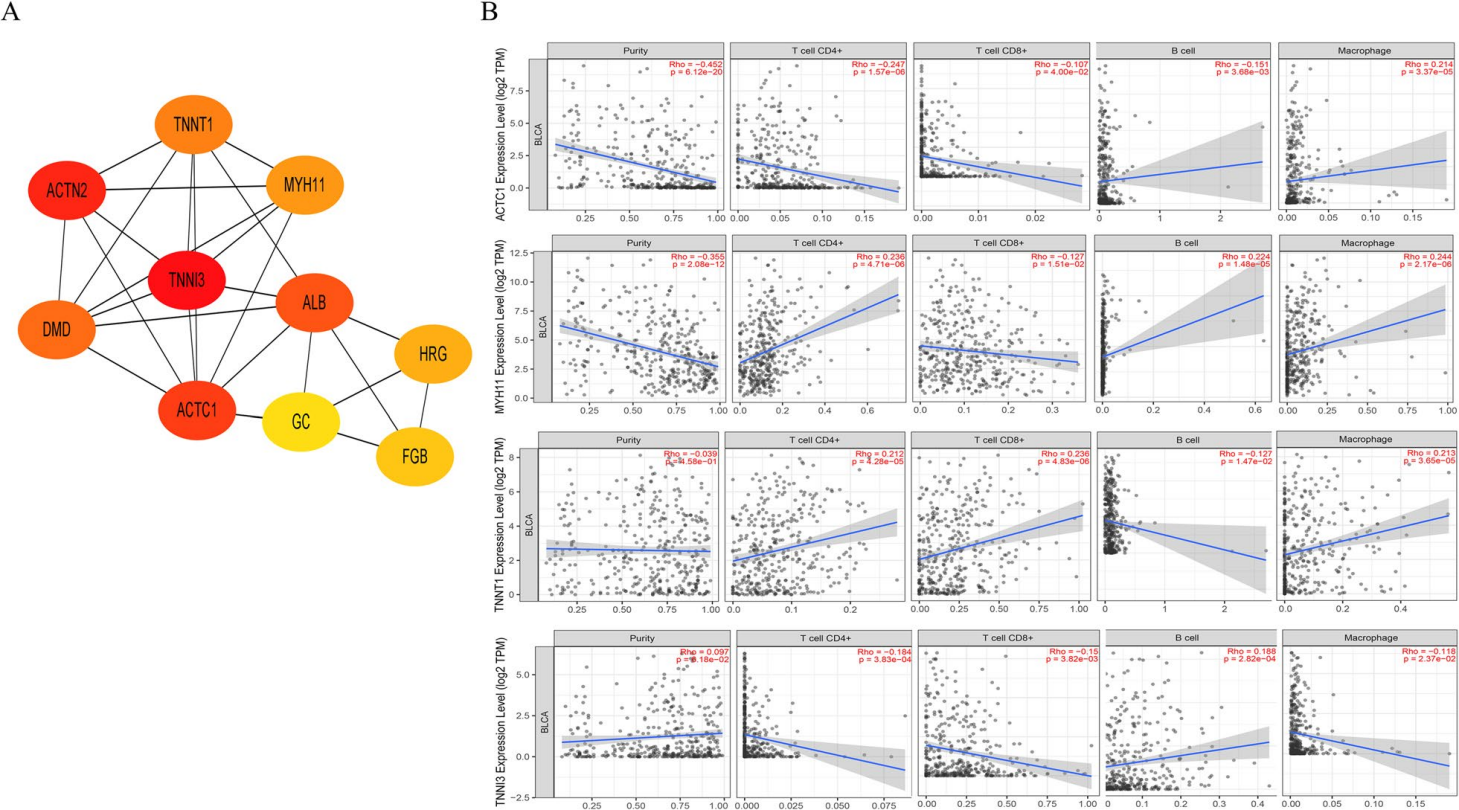
Immune infiltration analysis of key coding genes. **A**: Protein interaction network map of key coding genes. **B**: TNNI3, TNNT1, and ACTC1 and MYH11 coding gene expression and immune infiltration correlation analysis. The figure was made by STRING (STRING, version 11.5, Swiss Institute of Bioinformatics, Swiss) and TIMER (TIMER, version 2.0, Dana Farber Cancer Institute, US) online tools

Next, we used the WGCNA package to establish a co-expression network. First, a soft threshold value of 5 was selected (Fig. 8A). WGCNA analysis revealed that 71 genes were co-expressed with CYP4F8 (Fig. 8B), 80 with FAR2P1 (Fig. 8C), 346 with LINC01518 (Fig. 8D), 16 with LINC01764 (Fig. 8E), and 65 with DTNA (Fig. 8F, https://doi.org/10.6084/m9.figshare.20055986.v1). These co-expressed genes were sorted and de-duplicated for GO analysis. The main biological processes in which they were significantly enriched were muscle system processes and muscle tissue contraction (Fig. 9A, https://doi.org/10.6084/m9.figshare.20055989.v1). The cytological components were mainly contractile fibers and myogenic fibers (Fig. 9B, https://doi.org/10.6084/m9.figshare.20055992.v1). The molecular biological functions were mainly structural components of muscle and actin-binding (Fig. 9C, https://doi.org/10.6084/m9.figshare.20055995.v1). Enrichment analysis of the KEGG pathway mainly showed calcium signaling pathway and myocardial contraction, similar to the GO analysis findings (Fig. 9D). Following validation in the MsigDB gene set, enrichment analysis also revealed that these genes were associated with myocardial contraction and cardiac conduction (Fig. 9E). Using the STRING online tool, we found that six of the top 10 genes were associated with muscle contraction (Fig. 10A). Using the TIMER 2.0 online tool for immune infiltration analysis of these six genes, we found that the expression levels of TNNI3, TNNT1, ACTC1, and MYH11 were significantly correlated with the level of immune cell infiltration (Fig. 10B). Furthermore, we found that TNNT1, ACTC1, and MYH11 expression levels were negatively correlated with tumor purity, while TNNI3 expression levels were positively correlated with tumor purity.

## Discussion

The rapid development of industrialization worldwide has led to a continuous increase in the incidence of BC [7]. As a highly heterogeneous tumor with a poor prognosis and high recurrence rate [8], BC differs from other tumors of the urinary system due to its lack of good prognostic molecular markers [9]. Although the prognosis of BC is mainly related to histopathological staging [10], the use of modern genetic technologies is becoming more common. Thus, a diversified analysis combined with de novo tumor markers would be more conducive to accurate prediction. In recent years, numerous studies have found that lncRNAs are closely related to the prognosis of patients with urological tumors [11–14]. Therefore, an in-depth study of lncRNAs would be beneficial in identifying de novo markers associated with the prognosis of BC.

In the current study, we used different strategies to screen the TCGA database, and identified five genes associated with the prognosis of BC patients. Among them, DTNA was found to be an independent risk gene for the prognosis of BC patients, while CYP4F8, FAR2P1, LINC01518, and LINC01764 were identified as protective genes.

DTNA, as a member of the myotonic dystrophy protein family, is not only involved in calcium-binding and synapse formation and stability [15], but also plays a role in the progression of several malignancies as a proto-oncogene. For example, Fu et al. [16] found that miRNA-301b promotes the growth of esophageal cancer by regulating DTNA. In addition, Hu et al. [17] demonstrated that by binding to STAT3, DTNA further activates STAT3 resulting in the induction of TGF-β1 expression and inhibition of P53 expression, thereby promoting the progression of HBV-induced liver fibrosis, cirrhosis and hepatocellular carcinoma. Liu et al. [18] screened eight key lncRNAs, including DTNA, in early colon adenocarcinoma, and found that DTNA may be involved in the pathogenesis of early colon adenocarcinoma, as well as a potentially valuable tool for the diagnosis of early colon adenocarcinoma. Furthermore, using TCGA and GEO databases, Zhang et al. [19] successfully constructed and validated a new hypoxic signature model for BC, which accurately predicted the prognosis of BC patients. DTNA was identified as one of the key predictive genes in this model, consistent with our findings. The oncogenic role of DTNA may be associated with its involvement in the aggregation of nicotinic acetylcholine receptors, which control the synthesis and release of growth, angiogenesis and neurotrophic factors in cancer cells and the cancer microenvironment [20].

CYP4F8, a member of the cytochrome P450 family, functions as a 19-hydroxylase of prostaglandins in the seminal vesicles [21] and is involved in the metabolism of arachidonic acid [22]. When stimulated, arachidonic acid is converted by CYP4F8 into various biologically active arachidonic acids, which induce the proliferation of prostate cancer cells [23]. This was confirmed by Vainio et al. [24], who found that CYP4F8 was highly expressed in prostate cancer and was therefore a potential novel therapeutic target for prostate cancer. Other studies have shown that CYP4F8, a regulatory target of human peroxisome proliferator-activated receptor, has anti-angiogenic and anti-tumorigenic properties [25], which is consistent with the findings in our study.

The reprogramming of fatty acid metabolism in cancer can promote tumor growth, angiogenesis, survival, and metastasis [26]. The fatty acid metabolic pathway requires the generation of fatty acyl coenzyme A. FAR2P1, as a reductase of fatty acyl coenzyme A, may be involved in tumor cell progression through fatty acid metabolism. Wang et al. [27] also confirmed that FAR2P1 was highly expressed in the EGFR exon 19 deletion group of lung adenocarcinoma and that differential expression of FAR2P1 was associated with the development and progression of non-small cell lung cancer [28].

LINC01518 and LINC01764 are lncRNAs that act as targets or regulatory factors in multiple signaling pathways [29, 30]. Although their oncogenic roles have been studied in neuroblastoma [31], esophageal squamous cell carcinoma [32] and colorectal cancer [33], little is known about their anti-oncogenic roles.

In summary, in the current study, we identified five genes associated with BC malignancy. The differential expressions of CYP4F8, FAR2P1, LINC01518, LINC0176, and DTNA were verified in cell lines and tissues. In particular, the same trend was obtained in the different subtypes of BC. The overall expression levels of CYP4F8, FAR2P1, LINC01518, and LINC0176 were lower and the overall expression levels of DTNA were higher in patients with MIBC, when compared to patients with NMIBC. This indirectly suggested that CYP4F8, FAR2P1, LINC01518, and LINC0176 mainly played the role of anti-oncogenes and DTNA mainly played the role of oncogenes in BC patients. We found that a risk-prognosis model based on these five genes effectively assessed the survival of BC patients. Our findings confirmed that OS was worse in the high-risk group than the low-risk group. We further explored the potential biological functions of these five genes, using co-expression followed by GO analysis, and found that these genes were significantly associated with muscle composition, contraction, and calcium signaling pathways, which were further validated in the MsigDB gene set. Immune infiltration analysis revealed that the expression levels of TNNI3, TNNT1, ACTC1, and MYH11 were correlated with the level of immune cell infiltration and tumor purity. However, whether these four coding genes are related to the prognosis of BC patients and whether they are independently associated with the expression of the five lncRNAs will be addressed in future studies. In the current study, we also examined the TNNI3, TNNT1, ACTC1 and MYH11 genes, but unfortunately, none of these coding genes was associated with the prognosis of BC patients. In addition, we found no independent association between the four coding genes and the expression of the five lncRNAs in known databases using R software and the STRING online tool.

There are several limitations to the current study. First, the BC data in the TCGA database contain only one patient with squamous carcinoma and one patient with adenocarcinoma, while the remaining patients were all diagnosed with uroepithelial carcinoma. Similarly, all 165 samples in the GSE13507 dataset were from patients with uroepithelial carcinoma. Thus, due to the limited number of squamous and adenocarcinoma samples, differential analysis based on cancer type was not possible. Future studies should include analysis of additional public databases.Second, although this study demonstrated that DTNA, CYP4F8, FAR2P1, LINC01518, and LINC01764 could predict the occurrence and prognosis of BC, our study was limited to data mining analysis and qRT-PCR validation on cell lines and tissues, and did not include control or more in-depth validation studies.

## Conclusion

We identified five new genes by screening BC data in the TCGA database and studied their expression profiles, prognostic relevance, and biological functions. Our findings revealed that a prognostic model based on these five genes could predict the 1-, 3-, and 5-year OS in BC patients. Thus, our results indicate that these lncRNAs can predict the occurrence and prognosis of BC, and may be novel BC markers and potential therapeutic targets of BC that could prove to be beneficial in helping clinicians decide the most effective treatment strategy.

## Data Availability

All the data in the results of this study can be reasonably obtained from the corresponding authors. All data in the article is obtained from bioinformatics mining. The rawdata has been exposed on the https://figshare.com/ public resource platform, there are links in the article. There are no restrictions on data access in this article.

## Abbreviations

BC: Bladder cancer
GO: Gene ontology
HR: Hazard ratio
KEGG: Kyoto encyclopedia of genes and genomes
LncRNA: Long-stranded noncoding RNA
MIBC: Muscle invasive bladder cancer
ncRNAs: Non-coding RNAs
NMIBC: Non-muscle invasive bladder cancer
OS: Overall survival
PCA: Principal component analysis
qRT-PCR: Quantitative real-time polymerase chain reaction
ROC: Receiver operating characteristic curve
TCGA: The cancer genome atlas
WGCNA: Weighted gene co-expression network analysis
